# Early Dementia Questionnaire (EDQ): A new screening instrument for early dementia in primary care practice

**DOI:** 10.1186/1471-2296-14-49

**Published:** 2013-04-16

**Authors:** Zurraini Arabi, Noor Azah Aziz, Aznida Firzah Abdul Aziz, Rosdinom Razali, Sharifa Ezat Wan Puteh

**Affiliations:** 1 Department of Family Medicine, Faculty of Medicine and Health Sciences, Universiti Malaysia Sarawak, Lot 77, KTLD, Jalan Tun Ahmad Zaidi Adruce, 93150 Kuching, Sarawak, Malaysia; 2 Department of Family Medicine, Universiti Kebangsaan Malaysia Medical Centre, Kuala Lumpur, Malaysia; 3 Department of Psychiatry, Universiti Kebangsaan Malaysia Medical Centre, Kuala Lumpur, Malaysia; 4 Department of Community Health, Universiti Kebangsaan Malaysia Medical Centre, Kuala Lumpur, Malaysia

**Keywords:** Dementia, Early dementia questionnaire, Mini mental state examination

## Abstract

**Background:**

Worldwide, the population is ageing, resulting in an associated increase in dementia prevalence. Forgetfulness in elderly people is often perceived as normal in some local cultures and thus, the early detection of dementia in primary care requires detection of symptoms other than memory complaints.

This study was conducted to screen elderly patients for early dementia in primary care using a newly developed Early Dementia Questionnaire (EDQ) and comparing it with a standard assessment tool, the Mini Mental State Examination (MMSE).

**Methods:**

A cross-sectional study was conducted on a group of elderly patients using convenience sampling of consecutive patients. Elderly depression was excluded using the Geriatric Depression Scale (GDS). Exclusion criteria also included known cases of dementia. Inclusion criteria included a score of 5 or less in GDS and the presence of a reliable informant. A face-to-face interview was done using the EDQ with the patient and informant to elicit symptoms of early dementia. If the informant was not present, a telephone interview was used instead. The patient was then assessed with the Mini Mental State Examination (MMSE) using a cut-off point of 21.

**Results:**

Prevalence of dementia among 155 subjects was 52.3% by EDQ and 15.5% by MMSE. The EDQ demonstrated a sensitivity of 79.2% with specificity of 52.7%. Positive predictive value (PPV) of EDQ was 23.5% with the negative predictive value (NPV) of 93.2%. The strongest predictor of possible early dementia was complaints of memory problems (OR 26.22; 95% CI 2.03–338.14) followed by complaints of concentration problems (OR 14.33; 95% CI 5.53–37.12), emotional problems (OR 4.75; 95% CI 1.64–13.81) and sleep disturbances (OR 3.14; 95% CI 1.15-8.56). Socio-demographic factors, medical problems and smoking status were not associated with possible dementia (p>0.05), despite that 60–70% of the elderly had chronic illnesses.

**Conclusion:**

The EDQ is a promising alternative to MMSE for screening of early dementia in primary care.

## Background

Worldwide, there is an increase in the number of ageing population. By the year 2050, the global population of 60 and above is expected to increase to 2 billion in 2050 [1]. In Malaysia, the definition of elderly follows the standard document published in the ‘Policy for the Elderly in Malaysia’ which defined elderly population as those over 60 years of age, adopting the criteria set at the World Assembly on Aging in Vienna in 1982 [2].

One of the common conditions associated with ageing is dementia. It is defined as a progressive and largely irreversible clinical syndrome that is characterized by a widespread impairment of mental function [3]. As the population ages, the number of patients with dementia will continue to increase. According to the Global Burden of Disease from WHO document, (2003), it was estimated that 24.3 million people were living with dementia, and this condition contributed 11.2% of years lived with disability in people aged 60 and older [4].

Dementia causes a high burden of suffering to patients and their families. For the patients, it leads to increased dependency and complicates other co-morbid conditions [5]. For the families, caring for a person with dementia may lead to anxiety, depression and increased time spent caring for a loved one.

Although primary care is the first point of contact for elderly in the community, more than half of the elderly population who presented with mild and moderate dementia have never received the diagnosis of dementia [5,6]. The challenge in recognizing dementia in its early stage is that elderly people are often forgetful and thus this symptom is commonly perceived as a normal part of ageing in some local cultures. Early stage symptoms are commonly vague and less likely to present as overt memory problems [7]. Studies looking into the early signs of dementia found symptoms of disturbance of daily functioning, fixation on emotional events and disturbance of day-night rhythm as presentations of early stages of dementia other than the perceived loss of memories [7-9].

Dementia is most commonly diagnosed using the Mini Mental State Examination (MMSE) which is acknowledged as the gold standard for cognitive screening. MMSE is one of the most recognized and widely used instruments to assess for cognitive impairment and dementia in the elderly despite its limitations. [10] In the study done by Folstein et al. (1975), the cut of point of the score was 23 with the sensitivity of 100% and specificity of 44%. It is widely used in many other studies with the sensitivity ranging from 71.1% to 85.1% and specificity ranging from 81.3% to 95.6% depending on the study setting [10].

However MMSE has been known to possess its own limitations including its length, patient dependence, floor effect in advance dementia and ceiling effect in very mild disease [10]. Thus, those with a normal MMSE score may still have cognitive impairment and once the score is lower, the patient may already be in the later stage of the disease.

The factors of study setting, study populations and educational levels of the subjects are also known to affect the findings. Thus using MMSE alone as a tool for diagnosing dementia may be inadequate, as MMSE lacks sensitivity in assessing early features of dementia. Hence many cases might be missed until it is already in the later stage of the disease.

Although MMSE is known for its limitation in detecting early dementia, it is still the most widely used and studied worldwide. It is also often used as reference for comparative studies of other assessment tools [11]. In Malaysia, it is one of the commonest screening tools used in patients with suspected cognitive impairments in primary care clinics and hospital setting [12]. Thus, with these reasons MMSE was used as the gold standard in this study.

In Malaysia, the English version of MMSE was found not suitable due to language barriers and cultural differences. Malay version of MMSE has been translated and validated by Norlinah et al. (2009) in order to be used in our community. The optimal cut-off score were 21(sensitivity 88.5, specificity 75.3), 18 (sensitivity 97.1, specificity 90.0) and 17(sensitivity 97.7, specificity 93.3) [13].

There are other screening tools that can be used in primary care such the AD8, Mini-Cog and Montreal Cognitive Assessment (MoCA). The AD8 is an 8-item, informant questionnaire to assess whether there have been changes in certain areas of cognition and functioning in the past few years. If there is two or more ‘yes’ answer, then a patient is strongly suggestive of having dementia. The AD8 has a sensitivity and specificity of 85% and 86% respectively [14].

The Mini-Cog is a screening test with two components, three-item recall and clock drawing. A person is scored as demented if they recall none of the three words, or if they recall one or two of the three words and draw an abnormal clock [15]. When compared to MMSE, at a cutoff point of 25, the Mini-Cog had similar sensitivity (76% vs 70%) and specificity (89% and 88% for dementia) [16]. However, the disadvantage of the clock drawing test is in standardization of scoring which may lead to different interpretations

The Montreal Cognitive Assessment (MoCA) is a brief 30-questions test, developed to detect mild cognitive impairment, a clinical condition that often progress to dementia [17]. The scoring range from 0 to 30, with a score of 26 and higher is considered as normal. When compared to MMSE using a cutoff point of 26, the MoCA detected 90% of MCI subjects while the MMSE had a sensitivity of 18% to detect MCI. In the mild AD group, MoCA had a sensitivity of 100% while the MMSE had a sensitivity of 78%. Both MoCA and MMSE had a high specificity which was 87% and 100% respectively [18]. However, the conclusions regarding its validity can only be made in memory clinic settings [18] and studies in general practice is limited. It is also influenced by educational level of the patients.

Realizing the deficiency in identifying early dementia, this study aimed to develop a screening tool to recognize elderly patients who present in primary care setting with symptoms of early dementia other than presentation of memory complaints. The tool should also be less influenced by factors such as the educational level and cultural background. Most screening tools currently available, concentrate mainly on the cognitive functions, making them less suitable for screening of early dementia. Existing cognitive testing also requires extensive training of person conducting the testing to enable standardization of administration and scoring. This presents barriers to widespread use in primary care settings. Having an easily administered questionnaire that targets possible early symptoms of dementia will enable identification of patients who may not present with memory problems.

## Methods

The study was approved by the Medical research and Ethics Committee of the University Kebangsaan Malaysia.

### Sample population

This was a cross sectional study of elderly patients attending two primary care clinic in Cheras, Kuala Lumpur from the period May till October 2010 selected by convenience sampling of consecutive patients. Explanations were given and written informed consent was taken from them. Respondents who were aphasic, already diagnosed with dementia or depression and has severe hearing impairment were excluded from the study.

A total of 160 patients with their informants were recruited in this study. They were assessed for depression using Geriatric Depression Scale (GDS). Those who scored GDS ≤ 5 continued with the study. A total of 5 respondents who scored GDS > 5 was considered as having depression and was excluded. They were subsequently managed accordingly or based on the severity of their depression; further referred to a Psychiatrist.

The patient’s main informant was identified as this would be the person who would help in completing the questionnaire. A face to face or/ and phone interview were performed by the researcher with the patient and informant using the newly developed Early Dementia Questionnaire (EDQ). If there was any discrepancy between the patient’s and informant’s response, the response with a higher score for each question were taken for analysis. The patients were then reassessed using the Mini Mental State Examination (MMSE). The score obtained from the MMSE was compared with the scores obtained from the EDQ.

### Assessment tools

The questionnaires used in this study were:

#### Geriatric Depression Scale (GDS)

Geriatric Depression Scale (GDS) is a self reported scale used as a screening tool to assess depression among the elderly. GDS was used in this study as a tool to rule out pseudodementia due to depression before subjects were then assessed for early dementia using both EDQ and MMSE. The original version (GDS 30) consisted of 30 items in the form of yes/no responses and was designed for self-administration [19]. The shorter versions are GDS 15, GDS 10 and GDS 4. The validated Malay Geriatric Depression Scale-14 (M-GDS-14) is translated from GDS 15. At the cutoff point of 5/6, the M-GDS-14 detected all clinically significant depression with 95.5% sensitivity and 84.2% specificity [20]. In this study, the Malay Geriatric Depression Scale-14 with a cutoff point of 6 was used to detect depression.

#### Early Dementia Questionnaire (EDQ)

This questionnaire (Additional file 1) was developed based on combination of literature review, expert opinion and standardized assessment tools. The literature used for developing EDQ including De Lepeire et al., 1998 (for question1, 2, 6, 10, 11, 12, 13, 14, 16, 17, 18, and 20), Santacruz et al., 2001 (for question 5, 15 and 19) and Holzer et al., 2000 (for questions 4 and 9).

Expert opinions from specialists from UKMMC were obtained in 2 levels for the content validation. Firstly, consultations and discussions were done with the Family Medicine Consultants and Geriatric Psychiatrist for the content validation of the questionnaire. This is also to ensure that the questions accurately reflect the symptoms of early dementia. Secondly, a further 2 psychiatrists reviewed and analyzed the items in the questionnaire to determine the items tested were consistent with early dementia symptoms.

The standardized assessment tool used was the Informant Questionnaire on Cognitive Decline in the Elderly (IQCODE) [21] (for question 3, 7 and 8).

The aim was to identify symptoms presenting in local patients with early dementia. It is an interviewer guided questionnaire using face to face interview with the patient and informant.

The questionnaire was divided into three sections.

A) Patient/ Informant Identification

B) Socio-demographic data

C) Symptoms of early dementia

Symptoms of dementia were divided into 6 sub-domains; memory symptoms, concentration, physical symptoms, emotions, sleep disturbance and others. Memory symptoms (5 questions) were: checklist as memory support, difficulty in remembering events happening in the past 1 week (recent memory), unable to find kept/stored items and difficulty in remembering names/ familiar faces and familiar road directions. Concentration symptoms (4 questions) were: difficulty in following conversation, difficulty in understanding reading, difficulty in following stories on television and repetitive questioning. Physical symptoms (3 questions) were: difficulty carrying out daily house chores / work / hobby, difficulty in taking care of self / personal hygiene or using the toilet and disrupted movement (physical restlessness). Emotional symptoms (4 questions) were: unsuitable reaction towards external stimuli, obsession towards emotional events from the distant past, apathy or not interested in surroundings and looking for support/assurance from partner. Sleep disturbance (2 questions) were: night-day sleep rhythm disruption and restlessness at night. Other symptoms (2 questions) were: confusion after moving houses / in a new environment and outsiders aware of changes in term of behavior / appearance.

The questionnaire was originally developed in English and later translated into Malay language. The English version was translated to the Malay version (forward translation) by a bilingual professional translator and a medical officer. Subsequently, the Malay version was then back translated by two other bilingual professional translators and a medical officer. During the process of translation, only a few words had been changed from the original questionnaire to facilitate clarity.

### Test for reliability and validity of EDQ

Face validation of the questionnaire was done before the study was carried out. It involved 10 elderly patients who had fulfilled the inclusion criteria. Based on the feedback received, the questionnaire was later revised. The revised version was pretested again to a different group of 10 elderly patients. The final draft of the questionnaire was chosen for this study.

Statistical analysis was used to measure the reliability of the questionnaire using the SPSS version 12. The Cronbach’s Alpha for this questionnaire was 0.689 which was an acceptable internal consistency.

Scoring of EDQ was done through a Likert scale response ranging from 0–3. The score of 0 depicts never; 1 seldom; 2 sometimes and 3 always. The minimum score is 0 and the maximum score is 60. These were based on the symptoms a patient had in a week for the past 2 years. This was to determine the severity of the symptoms. Any discrepancy between the patient’s and informant’s response, the response with a higher score for each question were taken for total score. A score of 0–7 indicates that the patient is normal and a score of 8 or more indicates that the patient has possible early dementia.

To determine the cut off score for EDQ, a median score of 8 or more was used as indicative of possible dementia. This cut off was used because based on expert opinion and literature review, eight of the 20 questions of dementia symptoms in EDQ were identified as the earliest symptoms of dementia. These symptoms were:

•Require checklist as memory support

•Difficulty in remembering events that took place in the past 1 week (recent memory)

•Unable to find kept items

•Difficulty in remembering names / familiar faces

•Difficulty in remembering familiar road directions

•Difficulty understanding reading

•Obsession towards emotional event, although it has taken place long time ago (eg. death of family member or friend)

•Night-day sleep rhythm disruption

Analysis of the results in EDQ revealed that all the data for symptoms of dementia were not normally distributed with positive skewness value of 1.083, indicating that the data was clustered to the left. There was positive kurtosis value of 1.110 which indicated that the distribution was relatively peaked. Therefore, as a statistical rule, the symptoms of dementia were analyzed with non-parametric tests. Median instead of mean values was used for further statistical tests.

#### Mini Mental State Examination (MMSE)

For this study, the validated Malay version of MMSE-7 (serial 7) with cut-off level of 21 and below was used to detect possible dementia among elderly patients [13]. Those with scores lower than the cut-off scores indicate possible dementia. This cut off score was used as the study population was from urban and suburban area where it was postulated that majority of them would have some form of education.

### Statistical analysis

Data analysis was done using the Statistical Package for Social Science (SPSS) version 12.0. Descriptive analyses were used for demographic and categorical data. The variables shown to be associated with dementia were examined using bivariate analysis i.e. Chi-square test. The p value of less than 0.05 and confidence interval of 95% were considered as statistically significant.

## Results

One hundred and sixty (n=160) elderly respondents were initially approached to participate in the study. However, 5 respondents had GDS>5 thus were excluded from the study. Finally, 155 patients completed the study, giving the response rate of 96.9%. Seventy (45.2%) of the informants were interviewed by face to face while 85 (54.8%) were interviewed by phone.

Majority of the patients (89.0%) were from age group of 60–74 years with the median age of 67.0 years (IQR 63.0–71.0) with 56.8% of the respondents were male. The study populations’ ethnicities were as follows; Malay ethnicity contributed to 61.9%, Chinese at 31% and Indian at 7.1%. 80% of the patients were married, while widowed or divorced comprised of 18.1% and single respondents were only at 1.9%. The characteristics of the respondents are as described in Table 1.

**Table 1:** Sociodemographic and clinical characteristics of respondents

**Variables**	**Distribution of respondent**	**Percentages (%)**
	**( n=155)**	
	**Number(n)**	
**Age**		
60–64	62	40.0
65–69	43	27.7
70–74	33	21.3
75–79	13	8.4
≥ 80	4	2.6
**Gender**		
Male	88	56.8
Female	67	43.2
**Race**		
Malay	96	61.9
Chinese	48	31.0
Indian	11	7.1
**Marital status**		
Single	3	1.9
Divorced	1	0.6
Widowed	27	17.4
Married	124	80.0
**Education**		
No formal education	13	8.4
Primary	65	41.9
Secondary	63	40.6
Tertiary	14	9.0
**Occupation**		
Employed	17	11.0
Unemployed	48	31.0
Retired	90	58.0
**History of chronic illness**		
No	23	14.8
Yes	132	85.2
**History of hypertension**		
No	31	20.0
Yes	124	80.0
**History of dyslipidemia**		
No	64	41.3
Yes	91	58.7
**History of diabetes**		
No	91	58.7
Yes	64	41.3
**History of stroke**		
No	146	94.2
Yes	9	5.8
**History of smoking**		
Smoker	13	8.4
Nonsmoker	107	69.0
Former smoker	35	22.6

### Prevalence of dementia

The prevalence of early dementia using the newly designed EDQ in this study was 52.3%, whilst the prevalence of dementia using MMSE was 15.3%. The EDQ demonstrated a sensitivity of 79.2% with specificity of 52.7%. The positive predictive value (PPV) of EDQ was 23.5% with the negative predictive value (NPV) was 93.2%. The sensitivity and specificity of the MMSE were 88.5% and 75.3% respectively based from Norlinah et al., 2009.

There was no association between age, gender, educational level, living arrangement, occupation, medical problems and smoking status with possible dementia (p > 0.05) as shown in Table 2.

**Table 2:** Association between possible dementia (by EDQ) with variables

**Variables**	**Scoring from EDQ**		**χ**^**2**^	**p-value**
	**No dementia**	**Possible dementia**		
	**n (%)**	**n (%)**		
**Age (years)**				
60-64	30 (48.4)	32 (51.6)	1.927	0.749
65-69	23 (53.5)	20 (46.5)		
70-74	15 (45.5)	18 (54.5)		
75-79	5 (38.5)	8 (61.5)		
>80	1 (25.0)	3 (75.0)		
**Gender**				
Male	44 (50.0)	44 (50.0)	0.416	0.519
Female	30 (44.8)	37 55.2)		
**Educational Level**				
Low (no formal and primary)	37 (47.4)	41 (52.6)	0.032	0.984
Middle (secondary)	30 (47.6)	33 (52.4)		
High (tertiary)	7 (50)	7 (50.0)		
**Living Arrangements**				
With family	73 (48.3)	78 (51.7)	**-**	0.622
Alone	1 (25.0)	3 (75.0)		
**Occupation**				
Employed	9 (52.9)	8 (47.1)	0.538	0.764
Unemployed	21 (43.8)	27 (56.3)		
Retired	44 (48.9)	46 (51.1)		
**Medical problems (hypertension/DM/ hyperlipidemia)**				
No disease	11 (47.8)	12 (52.2)	0.301	0.860
Single disease	13 (43.3)	17 (56.7)		
Multiple disease	50 (49.0)	52 (51.0)		
**Stroke**				
Yes	2 (22.2)	7 (77.8)	-	0.171
No	72 (49.3)	74 (50.7)		
**Smoking Status**				
Smoker	3 (23.1)	10 (76.9)	**-**	0.083
Non smoker (never smoker and former smoker)	71 (50.0)	71 (50.0)		

Multivariate analysis (enter method) as shown in Table 3 demonstrated that four items in the EDQ significantly increased the likelihood of having early dementia as shown in Table 3. Those with **memory** problem were found to have an odds of **26.22** (95% CI: 2.03, 338.14) of having possible dementia than those without memory problems, controlling for other factors in the model. The odds of having possible dementia among those with **concentration** problems was **14.33** (95% CI: 5.53, 37.12) times higher than those without concentrations problems. Whereas the odds of having possible dementia among those with **emotional** problems was **4.75** (95% CI: 1.64, 13.81) and those with **sleep** problems was **3.14** (95% CI: 1.15, 8.56) times higher in likelihood to develop early dementia than those without emotional or sleep problems. This findings were statistically significant with p<0.05.

**Table 3:** Mutivariate logistic regression for predicting possible dementia

**Variables**	**Beta**	**Standard error**	**Wald**	**Df**	**p value**	**Exp (B)**	**95% Confidence interval**	
							**Lower**	**Upper**
**Memory**	3.266	1.305	6.268	1	**0.012**	26.216	2.033	338.137
**Concentration**	2.662	0.486	30.063	1	**0.000**	14.331	5.533	37.119
**Physical Symptoms**	0.541	0.535	1.021	1	0.312	1.718	0.602	4.905
**Emotions**	1.558	0.544	8.201	1	**0.004**	4.752	1.635	13.806
**Sleep disturbance**	1.143	0.513	4.966	1	**0.026**	3.135	1.148	8.564
**Others**	0.827	0.658	1.580	1	0.209	2.287	0.630	8.308

## Discussion

### Prevalence of dementia

**Figure 1:**
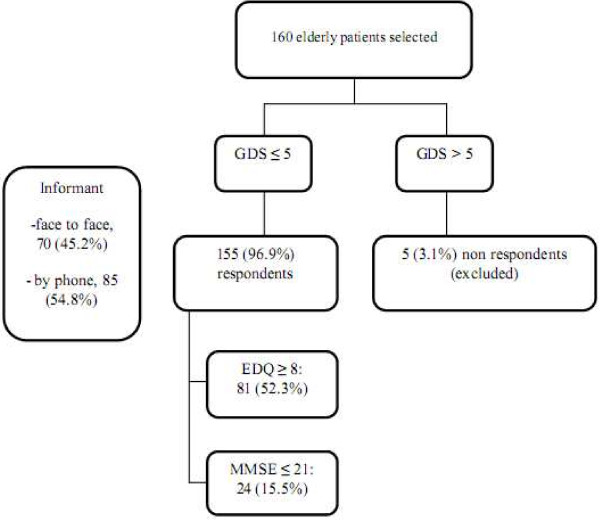
Results on prevalence of possible dementia using EDQ and MMSE.

The prevalence of dementia using EDQ was 52.3%, which was significantly higher than earlier studies [1,10,22,23] and when it was measured using the MMSE (Figure 1). The higher prevalence of dementia using EDQ could be because it concentrated on recognizing very early symptoms of dementia and it did not specifically exclude MCI which could present with similar symptoms.

As the information from EDQ was collected both from patients and informants, it is postulated that the recall was more accurate hence the higher prevalence obtained. This study was cross sectional where the study population was only screened with EDQ for the prevalence of early dementia. Thus, follow up assessments are needed to complete the diagnosis of dementia.

However, the EDQ might be useful in a primary care setting, as any score above 8 might alert the family physicians of the possibilities of early cognitive decline be it early dementia or any other neurological problems.

As expected, the prevalence of dementia using MMSE was much lower, documented at 15.5% (Figure 1). This difference of the prevalence could be due to the fact that MMSE commonly assesses patient’s cognitive function and decline in this parameter often occurs later in the course of dementia. The MMSE assessment is only proven accurate if patients complete the entire assessment. It also requires a certain level of understanding of numerical value or abilities in calculation. Hence, patients who are distracted or have overt dementia will not be able to answer the questions in MMSE properly. Although MMSE was used as the gold standard in this study, a complete diagnosis of dementia needs further assessment.

Earlier studies have also tried to overcome the shortcomings in the MMSE assessment tool by using different cut-off points in the MMSE for different levels of education [24] and population [25,26]. These studies used a two staged assessment with dementia diagnosis determined with a selected diagnostic tool.

It builds the rationale of using the EDQ as an alternative assessment for early dementia. The strength of EDQ is that it is not fully patient dependent hence, if a patient is not able to answer part of the questionnaire due to factors such as forgetfulness or distraction, the desired information can be obtained from the informant. Thus, it can provide a more accurate picture of dementia.

### The possible use of Early Dementia Questionnaire (EDQ) as a screening tool

For EDQ, the sensitivity was 79% and the specificity was 53%. PPV of EDQ was documented at 24%, thus 24% of those who scored 8 or more in EDQ have possible early dementia. However, the NPV of EDQ was 93%, thus 93% of those who scored less than 8 in EDQ do not have dementia.

With a sensitivity of 79% and PPV of 24%, EDQ generates about 47% of false positive rate. A high false positive rate is a characteristic of screening instruments used to detect low prevalence disorders [27]. Nevertheless, the high sensitivity reflects EDQ as a good screening tool for detection of early dementia where only about 20% of those with the disease in the population will be missed. However, the high NPV of 93% can accurately rule out early dementia in those who were screened negative with EDQ.

The advantages of EDQ are that it is simple, easily administered and is a user-friendly assessment tool. It can be easily and quickly used even in heavy clinic attendances in community setting. EDQ is also not fully patient-dependant as information can also be obtained from the patient’s informant. The high sensitivity signifies that EDQ is able to increase the likelihood of detecting an elderly person with subtle, early signs of dementia. Finally, EDQ is not influenced by education or cultural background of the patient, making it a feasible tool to be used across the community setting.

Being a new questionnaire, it has several disadvantages. The EDQ as a screening tool does not distinguish between patients with early dementia and those with mild cognitive impairment (MCI) who may present with similar presentation leading to high false positive rate. EDQ also relies heavily on the informant for information and this can be a barrier if the patient comes alone to the clinic and is unable to provide reliable contact information for an informant.

Although EDQ is not a diagnostic tool, a positive EDQ may alert the clinician to further assess the possibility of dementia. However, EDQ needs another validation study with a bigger sample for it to be validated properly.

### Symptoms associated with likelihood of having dementia from early dementia questionnaire (EDQ)

From the multiple regression analysis, out of the six sub-domains, four were noted to have statistically significant contribution to the EDQ model. These domains were memory, concentration, emotions and sleep. The strongest predictor of reporting possible dementia was memory (OR 26.22 (95% CI: 2.03, 338.14) followed by concentration, OR 14.33 (95% CI: 5.53, 37.12), emotions (OR 4.75 (95% CI: 1.64, 13.81) and sleep (OR 3.14 (95% CI: 1.15, 8.56). Thus, in patients with early dementia, it was 26 times more likely to have memory symptoms, 14 times more likely to have problems with concentration, 4 times more likely to have emotional symptoms and 3 times more likely to have sleep problems than those who did not have early dementia.

Memory symptoms are known from most literature as being part of the presentation of dementia. The finding regarding memory symptoms in this study further proves this information. The wide confidence interval might be contributed by the sample size and the sampling method used in this study. A study done by Lam LCW et al., 2005 found that memory complaints were useful in the assessment of patients with cognitive impairment as it correlated with objective performance of memory functions [28]. Subjective memory complaints also may predict dementia within 3 years especially if objective sign of memory deterioration is present together [29]. Although memory symptoms are a strong predictor, they may not be present in early dementia [30]. Thus, screening for other parameters is important in the detection of early dementia.

In primary care settings, patients come with a wide array of symptoms and diseases. Family physicians need to be efficient in the early detection of disease. No studies have looked into each symptom separately. Thus with this findings, patients who come with this symptoms can be further evaluated for cognitive impairment either dementia, MCI or other neurological problems. Patients can be monitored early for any deterioration.

Although dementia screening has been advocated to enable early intervention, it is still debatable whether screening is beneficial in terms of its impact to patients and most importantly the families. Decisions regarding transportation, living arrangements and overall care are not without negative effects. It is still in contention whether early recognition of dementia would burden the family and cause unnecessary stress to the carers. However, early detection of dementia would enable the patient and their carers to make decisions regarding transportation, living arrangements and other aspect of care at the time when patient is functioning at the highest possible level [6].

## Conclusion

EDQ can be viewed as a promising alternative to MMSE for screening of early dementia in primary care. However, EDQ needs another validation study with a bigger sample and includes other statistical analysis such as factor analysis, test- retest for it to be validated properly.

### Limitations

The study population was sampled from an urban and sub-urban clinic specific in Kuala Lumpur, thus the findings cannot be extrapolated to the general population. In collecting data, patients were asked to provide retrospective information, hence recall bias was unavoidable. EDQ was unable to distinguish between cases of MCI and other cognitive impairment thus giving rise to false positive dementia cases.

The prevalence of dementia was obtained by EDQ and MMSE only. A proper diagnostic evaluation using DSM IV criteria would be preferable to diagnose dementia but this would need more time to complete the study. However, these are preliminary findings of a new tool. We suggest future studies would subject all patients who screened positive with EDQ to undergo a proper diagnostic evaluation to confirm the diagnosis of dementia. Further study should include long term follow up to document the progression of the disease.

## Abbreviations

CI: Confidence interval; EDQ: Early dementia questionnaire; GDS: Geriatric depression scale; IQCODE: Informant questionnaire on cognitive decline in elderly; IQR: Interquartile range; MCI: Mild cognitive impairment; MMSE: Mini mental state examination; n: Number; NPV: Negative predictive value; OR: Odds ratio; P: Prevalence; PPV: Positive predictive value; SPSS: Statistical package for social science; UKMMC: University Kebangsaan Malaysia Medical Centre; WHO: World health organization; χ2: Chi square.

## Competing interest

The authors declared that they have no competing interest.

## Authors’ contribution

ZA conceived of the study, collected and analyzed the data, designed and draft the manuscript. ANA involved in the conception of the study, participated in the design of the study and helped to draft the manuscript. AFAA and RR participated in the design and coordination of the study. SEWP participated in the design of the study and assisted in the statistical analysis. All authors read and approved the final manuscript.

## Pre-publication history

The pre-publication history for this paper can be accessed here:

http://www.biomedcentral.com/1471-2296/14/49/prepub

## Supplementary Material

Additional file 1 Early Dementia Questionnaire (EDQ). Click here for file
