# Supplementary material for: Early Dementia Questionnaire (EDQ): A new screening instrument for early dementia in primary care practice
Source: BMC Fam Pract. 2013 Apr 16;14:49. doi: 10.1186/1471-2296-14-49 (PMC3637632; doi:10.1186/1471-2296-14-49)
Supplement: Additional file 1 — Early Dementia Questionnaire (EDQ). [file 1471-2296-14-49-S1.docx]

(For Office Use Only)

ID Code

**EARLY DEMENTIA QUESTIONNAIRE (EDQ)**

Thank you for participating in this project.

This study is to determine the presentation of early dementia. This is to enable us to identify the disease early to allow early treatment and intervention to be done at its early stage to prevent or delay the consequences of this disorder.

For each question, please choose the answer which applies to you and put a tick in the box which seems most applicable.

Some of the questions deal with personal matters. Information given is **confidential** and is used for the purpose of this study only.

1. **Patient / Informant Identification**
2. **Patient’s profile:**

Name: ________________________________________________

IC number: ____________________________________________

Telephone number: _____________________________________

Address: ______________________________________________

________________________________________________

________________________________________________

1. **Patient’s informant:**

Name: ________________________________________________

Relationship with patient: _________________________________

Telephone number: _____________________________________

1. **Socio-demographic data**

**Please answer by ticking the box that is most applicable.**

1. What is your age? _____________
2. Are you: Male Female
3. What is your ethnicity?

Malay

Chinese

Indian

Others Please state: __________

1. What is your marital status?

Single

Divorced

Widowed

Married

1. What is your educational level?

No formal education

Primary education (Standard 1-6)

Secondary education (Form 1-5)

Form 6, college, university

1. What is your occupation?

Employed please state: __________

Unemployed

Retired

1. What is your current living arrangement?

With family

Alone

With friends

Others please state: __________

8. Do you smoke?

Smoker

Nonsmoker

Exsmoker

1. Past medical history

Hypertension

Diabetes mellitus

Hyperlipidemia

Stroke

1. **Early stage dementia symptom**

**Please answer by marking ( √ ) in the most relevant space.**

|  | | **In a week**  **(since 2 years ago)** | | | | **Score** | |
| --- | --- | --- | --- | --- | --- | --- | --- |
|  |  | **Never**  **(0)** | **Seldom**  **(1)** | **Sometimes**  **(2)** | **Always**  **(3)** | **P** | **I** |
| **1.** | **A. Memory**  Require check list as memory support |  |  |  |  |  |  |
| **2.** | Difficulty in remembering events that took place in the past 1 week (recent memory) |  |  |  |  |  |  |
| **3.** | Unable to find kept item |  |  |  |  |  |  |
| **4.** | Difficulty in remembering names / familiar faces |  |  |  |  |  |  |
| **5.** | Difficulty in remembering familiar road directions |  |  |  |  |  |  |
| **6.** | **B. Concentration**  Difficulty in following conversation |  |  |  |  |  |  |
| **7.** | Difficulty understanding reading |  |  |  |  |  |  |
| **8.** | Difficulty following stories on television |  |  |  |  |  |  |
| **9.** | Repetitive questioning |  |  |  |  |  |  |
| **10.** | **C. Physical Symptoms**  Difficulty carrying out daily house chores / work / hobby |  |  |  |  |  |  |
|  |  | **In a week**  **(since 2 years ago)** | | | | **Score** | |
|  |  | **Never**  **(0)** | **Seldom**  **(1)** | **Sometimes**  **(2)** | **Always**  **(3)** | **P** | **I** |
| **11.** | Difficulty in taking care of self / personal hygiene or using the toilet |  |  |  |  |  |  |
| **12.** | Disrupted movement (physical restlessness) |  |  |  |  |  |  |
| **13.** | **D. Emotion**  Unsuitable reaction towards external stimuli (example: telephone ringing - emotional outburst) |  |  |  |  |  |  |
| **14.** | Obsession towards emotional event, although it has taken place long time ago (example: death of family member or friend) |  |  |  |  |  |  |
| **15.** | Apathy / no passion / not interested in surroundings |  |  |  |  |  |  |
| **16.** | Looking for support / assurance from partner |  |  |  |  |  |  |
| **17.** | **E. Sleep**  Night-day rhythm disruption |  |  |  |  |  |  |
| **18.** | Restlessness at night |  |  |  |  |  |  |
| **19.** | **F. Others**  Confusion after moving houses / in a new environment |  |  |  |  |  |  |
| **20.** | Outsiders aware of changes in term of behavior / appearance |  |  |  |  |  |  |

**P** = patient **I** = informant
